# Adapting field-mosquito collection techniques in a perspective of near-infrared spectroscopy implementation

**DOI:** 10.1186/s13071-022-05458-6

**Published:** 2022-09-26

**Authors:** Bernard Mouonniba Somé, Dari F. Da, Ruth McCabe, Nicaise Denis C. Djègbè, Lawata Inès Géraldine Paré, Kadidia Wermé, Karine Mouline, Thierry Lefèvre, Anicet Georges Ouédraogo, Thomas S. Churcher, Roch Kounbobr Dabiré

**Affiliations:** 1 Institut de Recherche en Sciences de La Santé, Direction Régionale, 399 avenue de la liberté, 01 BP 545, Bobo-Dioulasso 01, Burkina Faso; 2 Université Nazi Boni, Bobo-Dioulasso, Burkina Faso; 3 MRC Centre for Global Infectious Disease Analysis, Department of Infectious Disease Epidemiology, Imperial College London, London, W2 1PG UK; 4 Department of Statistics, University of Oxford, 24-29 St Giles, Oxford, OX1 3LB UK; 5 NIHR Health Research Protection Unit in Emerging and Zoonotic Infections, University of Liverpool, The Ronald Ross Building, 8 West Derby Street, Liverpool, L69 7BE UK; 6 Maladies Infectieuses et Vecteurs: Ecologie, Génétique, Evolution et Contrôle (MIVEGEC), IRD, CNRS, Montpellier University, Montpellier, France

**Keywords:** Near-infrared spectroscopy, *Anopheles*, Pyrethrum spray, *Plasmodium falciparum*, Chloroform

## Abstract

**Background:**

Near-infrared spectroscopy (NIRS) has the potential to be a useful tool for assessing key entomological parameters of malaria-transmitting mosquitoes, including age, infectious status and species identity. However, before NIRS can be reliably used in the field at scale, methods for killing mosquitoes and conserving samples prior to NIRS scanning need to be further optimized. Historically, mosquitoes used in studies have been killed with chloroform, although this approach is not without health hazards and should not be used in human dwellings. For the application of NIRS scanning it is also unclear which mosquito preservation method to use. The aim of the study reported here was to investigate the use of pyrethrum spray, a commercially available insecticide spray in Burkina Faso, for killing mosquitoes

**Methods:**

Laboratory-reared *Anopheles gambiae* and *Anopheles coluzzii* were killed using either a pyrethrum insecticide spray routinely used in studies involving indoor mosquito collections (Kaltox Paalga®; Saphyto, Bobo-Dioulasso, Burkina Faso) or chloroform (“gold standard”). Preservative methods were also investigated to determine their impact on NIRS accuracy in predicting the species of laboratory-reared *Anopheles* and wild-caught mosquito species. After analysis of fresh samples, mosquitoes were stored in 80% ethanol or in silica gel for 2 weeks and re-analyzed by NIRS. In addition, experimentally infected *An. coluzzii* and wild-caught* An. gambiae* sensu lato (s.l.) were scanned as fresh samples to determine whether they contained sporozoites, then stored in the preservatives mentioned above for 2 weeks before being re-analyzed.

**Results:**

The difference in the accuracy of NIRS to differentiate between laboratory-reared *An. gambiae* mosquitoes and *An. coluzzii* mosquitoes killed with either insecticide (90%) or chloroform (92%) was not substantial. NIRS had an accuracy of 90% in determining mosquito species for mosquitoes killed with chloroform and preserved in ethanol or silica gel. The accuracy was the same when the pyrethrum spray was used to kill mosquitoes followed by preservation in silica gel, but was lower when ethanol was used as a preservative (80%). Regarding infection status, NIRS was able to differentiate between infected and uninfected mosquitoes, with a slightly lower accuracy for both laboratory and wild-caught mosquitoes preserved in silica gel or ethanol.

**Conclusions:**

The results show that NIRS can be used to classify *An. gambiae* s.l. species killed by pyrethrum spray with no loss of accuracy. This insecticide may have practical advantages over chloroform for the killing of mosquitoes in NIRS analysis.

**Graphical Abstract:**

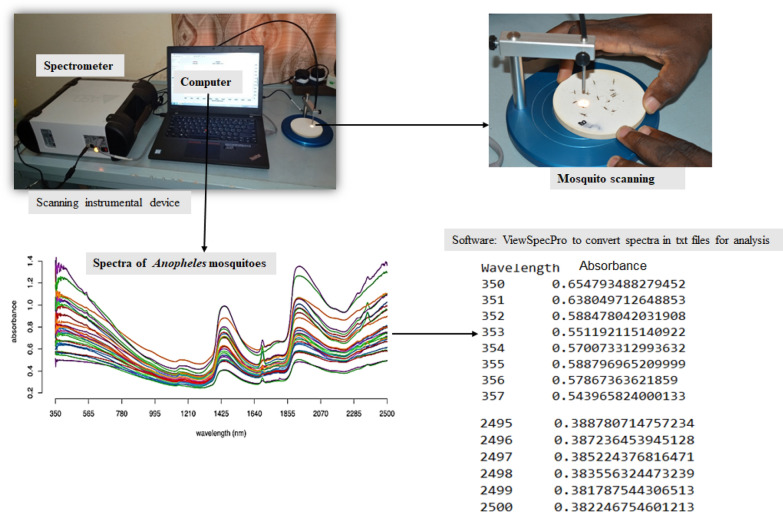

**Supplementary Information:**

The online version contains supplementary material available at 10.1186/s13071-022-05458-6.

## Background

Mosquito control is one of the most important global public health interventions in the fight against diseases such as malaria, dengue, chikungunya and zika, among others [1, 2]. Entomological monitoring is an important scientific and routine surveillance tool although commonly used methods, such as the determination of mosquito species using molecular methods such as PCR are technically laborious, require expensive reagents and qualified workers and are often time consuming. These disadvantages often mean that only a limited number of specimens can be processed, reducing the applicability of the data generated.

Near-infrared spectroscopy (NIRS) is a rapid, high-throughput and relatively inexpensive technique that has been used for a decade to predict the species, age and infection status of certain disease vectors, such as *Anopheles* and *Aedes* mosquitoes [3–5]. Many of these different studies used laboratory or field mosquitoes to assess the accuracy of NIRS to differentiate between *Anopheles* species [3, 4], mosquito age [3, 6] and *Anopheles* infection status using *Plasmodium* culture [7, 8]. The reliability of the method for application in entomological surveillance studies using wild mosquitoes is less clear, with models trained on laboratory-reared mosquitoes being unable to accurately predict the infection status or age of field mosquitoes [5, 9]. Further work is therefore required to verify these results, and it may be necessary to calibrate NIRS models with greater numbers of wild-caught mosquitoes. Model calibration requires a large number of samples to improve accuracy [6]. Reliable surveillance will also require a large number of mosquitoes to be processed in order to overcome sampling heterogeneity [6]. However, a number of practical issues, such as the collection method (pyrethrum spray catch, human landing catch, residual fauna catch, etc.) of *Anopheles* from the field as well as the preservation of the collected samples for NIRS processing in the laboratory, must be addressed before this technique there can be more widespread implemented. Historically, mosquitoes for NIRS analysis have been killed with chloroform, which must be handled with great care. Chloroform is toxic and carcinogenic upon inhalation [10]. Therefore, researchers should be working in a well-ventilated room in the laboratory when it is used to kill mosquitoes. This toxicity limits its applicability for use in human residences when the aim is to catch wild mosquitoes and other insects.

To address these shortcomings, we set out to explore a more practical solution for mosquito killing. Kaltox Paalga® (Saphyto, Bobo-Dioulasso, Burkina Faso) is a commercial insecticidal product commonly used in Burkina Faso for killing insects or collecting indoor mosquitoes (i.e. pyrethroid spray catches [11]) for use in various studies. This pyrethrum spray is a publicly available health product registered in Burkina Faso. It is classified in toxicity class U in the WHO classification system for pesticides, which means it does not constitute the same risk during normal use as chloroform. Thus, the first objective of our study was to investigate whether a pyrethrum insecticide can replace chloroform for killing mosquitoes to be analyzed by NIRS.

An NIR spectrometer is relatively portable, but it does require a reliable power source. In terms of practicality, NIRS may be easier if the spectrometer can be kept in a central location and preserved samples are transported from the collection site to the laboratory where they can be processed. Previous studies on species identification or mosquito age grading have used samples preserved in RNAlater® reagent or by refrigeration or freezing [12, 13]. However, these preservation methods are not practical in low-income countries, as they are expensive, require basic laboratory conditions for storage and are not widely available in sub-Saharan Africa. Desiccation of samples in silica gel has also shown to be a good preservation method [13], but some specific analytical techniques, such as mosquito dissection, cannot be performed following desiccation, thereby reducing the usefulness of the sample for determining other useful entomological parameters such as parasite load or parity status. Ethanol, a relatively cheaper preservative that is readily available in laboratories throughout Africa, could be a more economic field mosquito preservative for use with NIRS. Hence, our second objective was to test the impact of preserving *An. gambiae *sensu lato (s.l.) in ethanol or silica gel on NIRS accuracy. NIRS accuracy in these two objectives was assessed by the ability of this technique to differentiate between *Anopheles gambiae *sensu stricto (s.s.) and *Anopheles coluzzii*, two closely related mosquito species which are the primary vectors for malaria transmission in Burkina Faso [14]. The effect of the killing and preservation methods on the ability of NIRS to determine whether mosquitoes were infected or not with *Plasmodium falciparum* sporozoites was also evaluated.

## Methods

### Study design

The study was conducted in Burkina Faso at the “Institut de Recherche en Science de la Santé” (IRSS), Bobo-Dioulasso, with institutional ethic committee approval (Reference no.: A018-2017/CEIRES). Two experiments were performed to assess NIRS accuracy in predicting either the *Anopheles* species or their *Plasmodium* infection status. We first explored the influence of mosquito collection using the pyrethrum insecticide as the killing agent on NIRS accuracy to predict *Anopheles* species. The killing efficacy of the pyrethrum insecticide was compared to that of chloroform (the gold standard killing method for NIRS analyses) using laboratory colonies of *An. gambiae* and *An. coluzzii*. Laboratory-reared *An. gambiae* and *An. coluzzii* were obtained from an outbred colony established [15] in 2015 and 2016, respectively, and routinely identified by conventional PCR assays [16]. These two *Anopheles* species were repeatedly replenished with F_1_s from wild-caught female mosquitoes. We then explored the best way to preserve *Anopheles* samples for NIRS future analysis, testing 80% ethanol and silica gel as preservatives for both laboratory-reared and wild-caught mosquitoes. Field mosquitoes were collected inside of human dwellings in Longo village (11°34′57″N, 4°33′27″W), located about 60 km from Bobo-Dioulasso.

### Killing methods for determining *Anopheles* species

**Fig. 1:**
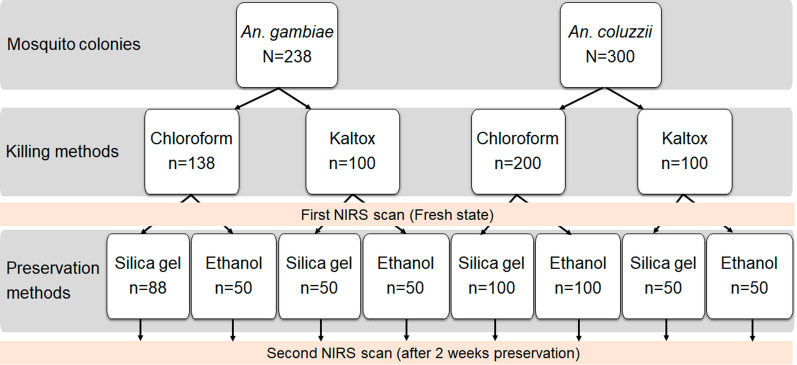
Summary of the experimental design and sample size of laboratory mosquitoes scanned to determine NIRS accuracy to predict *Anopheles* species according to killing and preservation methods. For the model trained to predict the effect of the Kaltox (Kaltox Paalga® insecticide) killing method, 100 *Anopheles* mosquitoes (50 *An. gambiae* and 50 *An. coluzzii*) were used. Validating and testing the model were realized using 50 *Anopheles* mosquitoes per subset (25 *An. gambiae* and 25 *An. coluzzii*). When training the model to predict the effect of the chloroform killing method, 138 *Anopheles* mosquitoes (68 *An. gambiae* and 68 *An. coluzzii*) were used per replication. This model was validated and tested using each time 68 *Anopheles* mosquitoes (34 *An. gambiae* and 34 *An. coluzzii*) per data subset. NIRS, near-infrared spectroscopy

Laboratory-reared *An. gambiae* and *An. coluzzii* were used to test a pyrethrum insecticide as the mosquito killing agent for NIRS analysis. The insecticide chosen, Kaltox Paalga®, is a combination of pyrethroids (allethrin, 0.27%; permethrin, 0.17%; tetramethrin, 0.20%), an organophosphorus (chlorpyrifos ethyl, 0.75%) and solvent in an aerosol formulation of 98.61%. For this study, 7-day-old *An. gambiae* and *An. coluzzii* females were divided into two groups, and each group was assigned to be killed using either pyrethrum insecticide (Kaltox Paalga®) or chloroform vapor (currently used gold standard) for NIRS analysis to predict *Anopheles* species (Fig. 1). Freshly killed mosquitoes were scanned using NIRS (referred to as “fresh state” mosquitoes) before being assigned to the appropriate preservation group.

### Mosquito preservation methods for determining *Anopheles* species and their *P. falciparum* infection status

#### Determining mosquito species

The same laboratory-reared *Anopheles* killed using either chloroform or the pyrethrum spray as described above were divided into two groups, with the mosquitoes of one group of *Anopheles* stored individually in 200 µl of 80% ethanol in Eppendorf tubes and those of the second group individually desiccated with silica gel in Eppendorf tubes as described in previous studies [12, 17]. The experimental design and the samples sizes used for each group are summarized in Fig. 1.

**Fig. 2:**
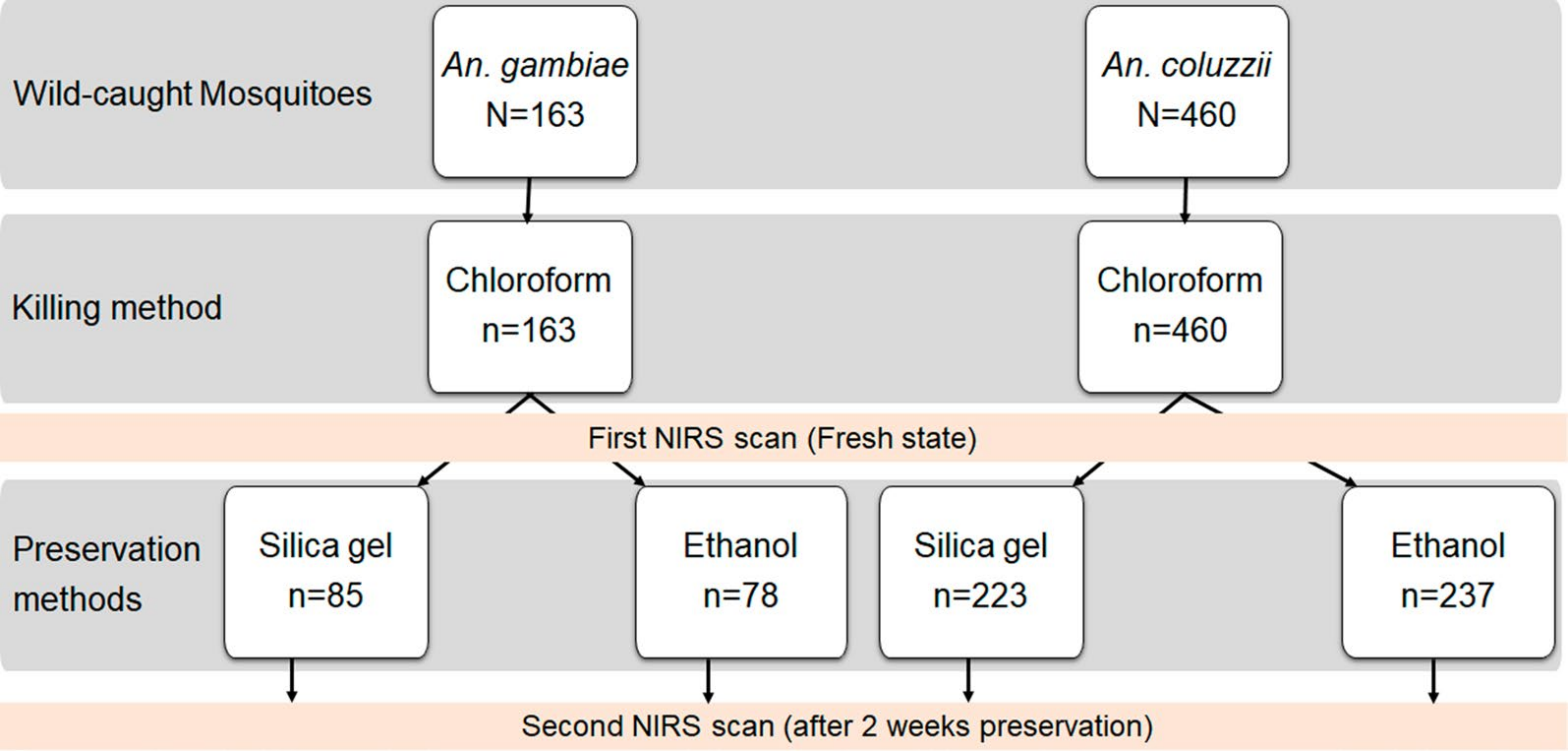
Summary of the experimental design and sample size of wild-caught *Anopheles* scanned to determine NIRS accuracy for predicting *Anopheles* species according to preservation methods. Using silica gel as preservative, the model was trained taking into account 85 *Anopheles* mosquitoes (42.5 *An. gambiae* and 42.5 *An. coluzzii*). The trained model was validated using 42 *Anopheles* and tested on 42 *Anopheles* (21 *An. gambiae* and 21 *An. coluzzii*). With ethanol as preservative, the model was trained using 78 *Anopheles* mosquitoes (39 *An gambiae* and 39 *An. coluzzii*) per replication. This model was validated using 39 *Anopheles* and tested on 39 *Anopheles* mosquitoes (19.5 *An. gambiae* and 19.5 *An. coluzzii*)

In addition to the laboratory specimens, wild-caught *An. gambiae* s.l. mosquitoes killed using chloroform only were immediately scanned by NIRS and stored in either ethanol or silica gel for future analysis with NIRS to predict *Anopheles* species (Fig. 2).

All samples were stored at the insectary (27 °C ± 2; 70% ± 10 relative humidity; 12:12-h light:dark conditions) for 2 weeks, then scanned for a second time. *Anopheles* preserved in ethanol were exposed to air on a paper towel for about 5 min to allow ethanol evaporation prior to NIRS scanning of individual mosquitoes. The cephalothorax of wild-caught mosquitoes was analyzed by conventional PCR to determine *Anopheles* species using a common protocol [16].

#### Detecting* P. falciparum* infection status of mosquitoes

The potential impact of preservation method on the ability of NIRS to determine whether *Anopheles* were infected with *P. falciparum* was explored using laboratory-reared *An. coluzzii* experimentally infected with *P. falciparum* and wild-caught mosquitoes.

**Fig. 3:**
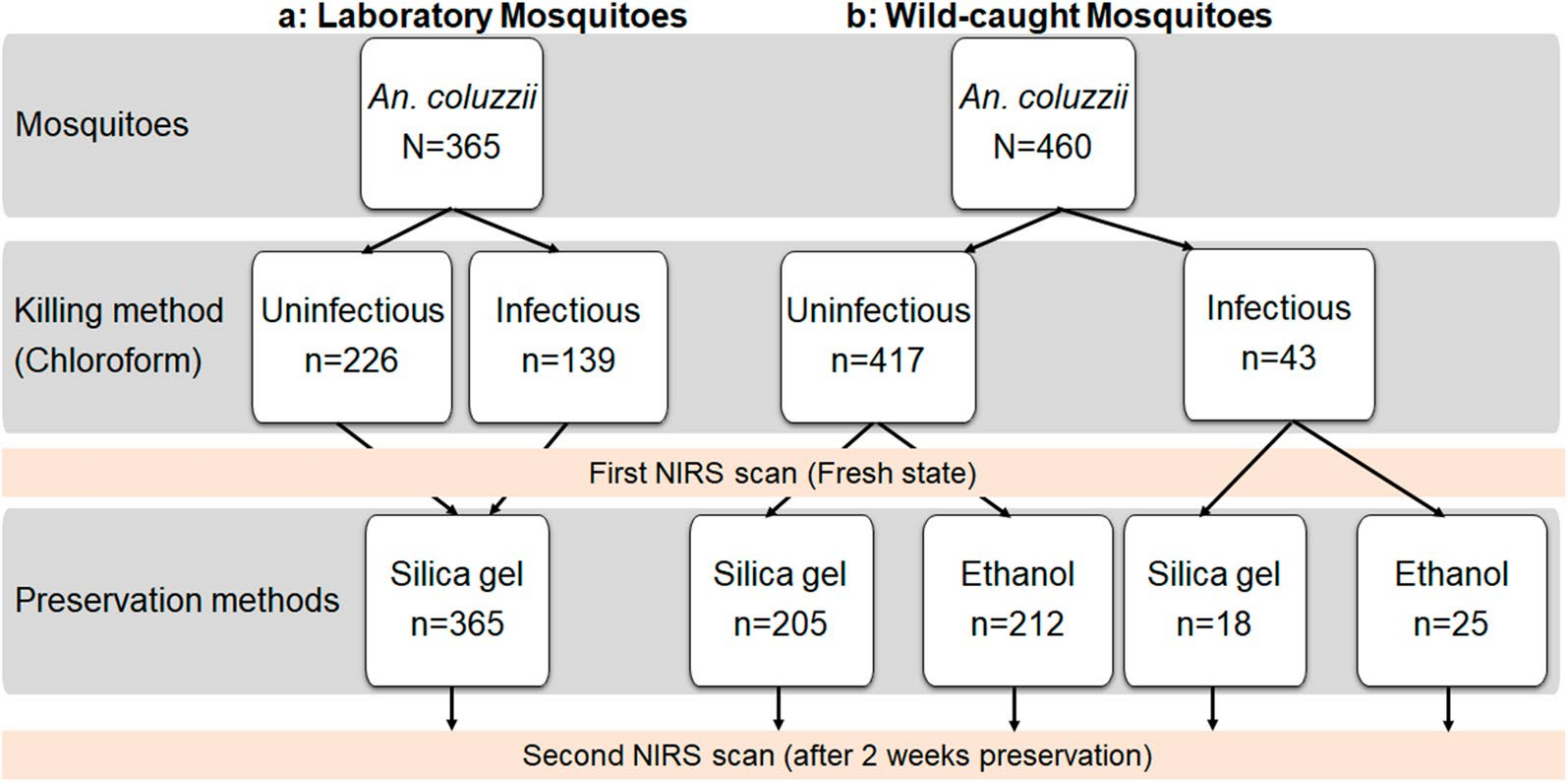
Summary of the experimental design and sample size of laboratory-reared (a) and wild-caught (b) *Anopheles* scanned to determine the accuracy of NIRS to predict *Anopheles*-*Plasmodium* infectious status according to preservation method. In the laboratory, the model was trained to predict *Plasmodium* infection status of* An. coluzzii* using 139 *An. coluzzii* mosquitoes (69.5 uninfected and 69.5 infected) per replication. This model was validated with 68 *Anopheles* and used to test 68 *Anopheles* (34 uninfected and 34 infected). To predict mosquito infection status in the field using ethanol as preservative, the model was trained using 25 *An. coluzzii* mosquitoes (12.5 uninfected and 12.5 infected) per replication. Validating and testing the model were realized with 12.5 *Anopheles* mosquitoes per data subset. With silica gel, the model was trained using 18 *An. coluzzii* mosquitoes (9 uninfected and 9 infected) per replication. Validating and testing the model were realized with 9 *Anopheles* mosquitoes per data subset

Three-day-old laboratory-reared female *An. coluzzii* mosquitoes were infected with natural isolates of *P. falciparum* using the direct membrane feeding assays protocol [18]. Blood-fed *Anopheles* were kept at the IRSS insectary and fed with 10% glucose solution ad libitum for 2 weeks, which was the period of time estimated to be needed to obtain infected *Anopheles* (sporozoite stage), and then killed with chloroform for analysis using NIRS. Following this first analysis, the mosquitoes were individually desiccated with silica gel in Eppendorf tubes for 2 weeks and analyzed again using NIRS (Fig. 3a).

Wild-caught mosquitoes were collected early in the morning in the living rooms of human dwellings in Longo village using mouth aspirators, as previously demonstrated by Anthony et al*.* [19]. Once transported to the IRSS laboratory, all *An. gambiae* s.l. females were maintained under standard insectary conditions (27 °C ± 2; 70% ± 10 relative humidity 12:12-h light:dark conditions) and fed with 10% glucose solution for 7 days for NIRS analysis. The 7-day period is considered to be appropriate for complete blood digestion to avoid any interference by blood on mosquito spectra during NIRS scanning and also to allow some sporozoites to complete maturation. *Anopheles gambiae* s.l. were killed using chloroform immediately prior to being analyzed by NIRS and then preserved individually in 80% ethanol or desiccated with silica gel in Eppendorf tubes. The mosquito samples were stored at the insectary for 2 weeks before being analyzed by NIRS for the second time.

After the second analysis by NIRS, the cephalothorax of each experimentally infected *Anopheles* and wild-caught mosquito was analyzed using quantitative PCR (qPCR) to determine their *P. falciparum* infection status targeting the mitochondrial gene that codes for cytochrome* c* oxidase (Cox1) [20]. After DNA extraction using DNAzol (Invitrogen, Thermo Fisher Scientific, Waltham, MA, USA), primers qPCR-PfF (5′-TTACATCAGGAATGTTATTGC-3′) and qPCR-PfR (5′-ATATTGGATCTCCTGCAAAT-3′) were used for amplification. A sample reaction occurred in a total volume of 10 μl containing 1 μl of DNA (approx. 40 ng/μl), 4.6 μl of water, 2 μl of 1× HOT Pol EvaGreen qPCR Mix Plus ROX (Solis BioDyne, Tartu, Estonia) and 1.2 μl of each primer at 5 μM). PCR cycling consisted of an activation step at 95 °C for 15 min, followed by 40 cycles of denaturation at 95 °C for 15 s, and a final renaturation/extension step at 58 °C for 30 s.* Anopheles *female mosquito samples were considered to be positive for *P. falciparum* at qPCR showed a threshold cycle (Ct) < 35 and > 75 and melting temperature (Tm) < 80 °C.

Conventional PCR was performed to determine the infection status of wild-caught *Anopheles* species [16]. Similar to the laboratory experimental infection, wild-caught *An. coluzzii* was the only species used to determine mosquito infection status by NIRS (Fig. 3b).

### Mosquito scanning and data analysis

Mosquitoes were scanned using a LabSpec4 Standard-Res *i* (standard resolution, integrated light source) NIR spectrometer and a bifurcated reflectance probe mounted 2 mm from a spectral on white reference panel (ASD Inc., Malvern PANalytical, Longmont, CO, USA). Absorbance was measured at a wavelength of 2151 nm in the interval 350–2500 nm of the electromagnetic spectrum. All specimens were scanned on the side under the focus of the light probe, and spectra were recorded with RS3 spectral acquisition software (ASD Inc., Malvern PANalytical) that automatically records the average spectra from 20 scans.

A previously published statistical machine learning approach was used to fit and cross-validate the best model using a generalized linear model [9, 21]. A binomial logistic classification model was used to determine *Anopheles* species (*An. gambiae* or *An. coluzzii*) and two response classes were assigned: *y* = 0 for *An. gambiae* and *y* = 1 for *An. coluzzii*. Partial least-squares regression methods were used implemented in a specifically designed R package (downloaded from https://github.com/pmesperanca/mlevcm and described in [21]) (for code, see Additional file 1: Dataset S1; Additional file 2: Dataset S2). Simple models without spectra smoothing or penalized estimation of the coefficient function were used for all analyses. Data are split (at a ratio of 2:1:1) into three subsets for model training, validation (where the number of principal components are selected, varying between 2 and 50) and testing (which estimates the generalized error). In all models, observations were balanced by random sampling from the total number of scans in the class to ensure an equal number of observations per class. These data subletting, training, validation and testing steps were repeated 100 times, and models were averaged over each realization. Fisher’s exact test was used to test for statistically significant differences between the accuracies of the different models. All data analyses were performed using R software version 4.0.2 ® Foundation for Statistical Computing, Vienna, Austria).

As noted above, models were calibrated using mosquitoes subjected to the same killing and preservation methods. For example, fresh mosquitoes were not used to predict the species or *Plasmodium* infection status of ethanol-preserved mosquitoes.

## Results

### Killing methods for NIRS analysis

**Fig. 4:**
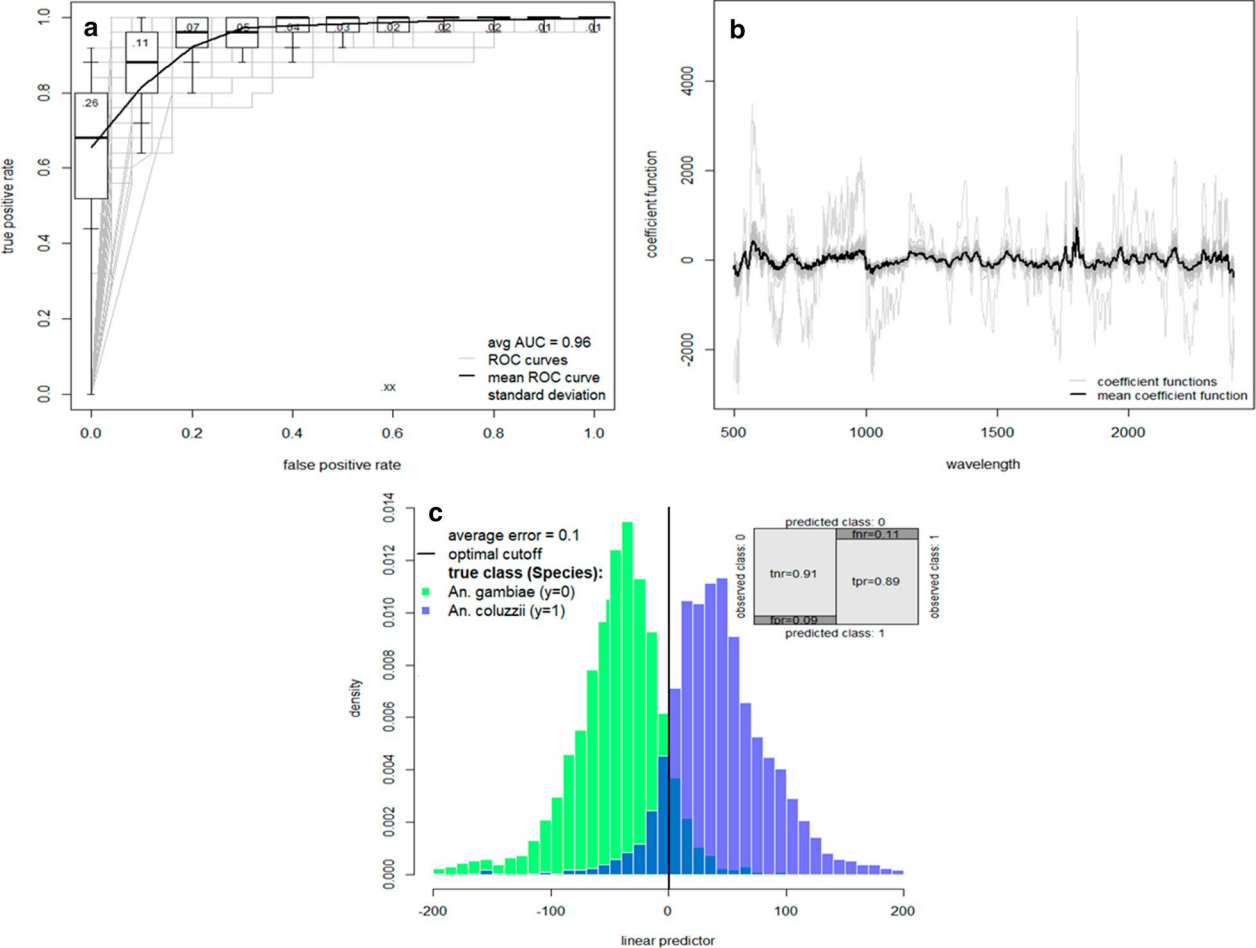
NIRS ability to predict laboratory-reared mosquito species killed with Kaltox. **a** The ROC curve showing the false positive and true positive rates for the different classification probability thresholds, with the overall performance given by the average AUC. **b** Coefficient functions for each of the 100 dataset randomizations (gray lines) and the corresponding average (black line). **c** Histogram of the estimated linear predictor for the test mosquitoes, with the color of the bars indicating the true class, shows the model’s ability to separate the two groups of mosquitoes. The vertical black line indicates the optimum threshold for classifying mosquitoes as *An*. *gambiae* or *An*. *coluzzii*. The shaded area where the two distributions overlap corresponds to misclassified test observations, with false negatives to the left of the optimal classification threshold and false positives to the right. The confusion matrix (inset) shows the different error rates: false negative rate (fnr), false positive rate (fpr), true negative rate (tnr; *An. gambiae*); true positive rate (tpr; *An. coluzzii*). AUC, Area under the ROC curve; ROC, receiver operating characteristic

A total of 538 *Anopheles* aged 7 days were used to assess the accuracy of NIRS for determining the species of *Anopheles* mosquitoes killed using pyrethrum spray or chloroform (sample sizes are given in Fig. 1). The average spectra for these two killing options differed slightly (Additional file 3: Fig. S1). The binomial classification model trained with mosquitoes killed using chloroform correctly classified *An. gambiae* and *An. coluzzii* analyzed in the fresh state with 92% accuracy (*An. gambiae*, 94%; *An. coluzzii*, 90%). The same trend of NIRS accuracy (90%) was obtained for mosquitoes killed with pyrethrum spray, with NIRS able to classify mosquitoes as *An. gambiae* or *An. coluzzii* with 91% and 89% accuracy, respectively (Fig. 4). Fisher’s exact test showed that there was no difference in the accuracy of NIRS to differentiate *An. gambiae* s.l. species killed by chloroform and those killed by pyrethrum spray (*P*-value = 0.433). These findings indicate that pyrethrum spray catches could be used as an alternative process to kill mosquitoes for NIRS analysis.

### Accuracy of NIRS to predict *Anopheles* species after preservation

The average spectra of each killing and preservation method are shown in Additional file 4: Fig. S2. Globally, a distinctive difference was observed between the average spectra obtained from mosquitoes freshly analyzed and the average spectra obtained from *Anopheles* preserved in silica gel or in ethanol. However, mosquito NIRS spectra were more influenced by the silica gel than by ethanol. NIRS was able to distinguish between laboratory-reared *An. gambiae* and *An. coluzzii* after preservation in silica gel or 80% ethanol. The average accuracy ranged between 83% and 94% depending on the mosquito killing process and the preservation methods for each species (Table 1). The overview comparison revealed that there was no substantial difference in the accuracy of NIRS to predict *Anopheles* species in the fresh samples and the preserved ones (Table 2).

**Table 1:** Accuracy of near-infrared spectroscopy to predict each species of the* Anopheles gambiae *sensu lato complex according to killing option and preservative method

Killing process	Preservative	Accuracy at predicting *Anopheles* species					
		*An. gambiae *sensu stricto			*An. coluzzii*		
		Fresh (%)	Preserved (%)	*P*	Fresh (%)	Preserved (%)	*P*
Chloroform	Silica gel	91	93	0.781	92	88	0.356
	80% Ethanol	92	92	1	89	88	1
Kaltox®	Silica gel	91	94	1	83	86	1
	80% Ethanol	92	89	1	88	85	1

*P* > 0.05 (Fisher's exact test) indicates no difference between the accuracy of near-infrared spectrometry (NIRS) before and after mosquito preservation

**Table 2:** Overview of near-infrared spectroscopy accuracy in predicting *An. gambiae* sensu lato species according to killing option and preservative method

Killing process	Preservative	*Anopheles* species		
		Specificity (*An. gambiae* sensu lato) (%)	Sensitivity (*An. coluzzii)* (%)	Accuracy (%)
Chloroform	Fresh all	94	90	92
	Silica gel	93	88	90
	80% Ethanol	92	88	90
Kaltox®	Fresh all	91	89	90
	Silica gel	94	86	90
	80% Ethanol	89	85	87

Given these optimistic results derived using laboratory-reared *Anopheles*, we then focused on determining whether these preservation methods could be extrapolated to field conditions. A total of 731 wild-caught mosquitoes killed using chloroform vapor were immediately scanned by NIRS, preserved in either 80% ethanol or silica gel and then re-scanned 2 weeks later. The main *Anopheles* species identified by qPCR were *An. coluzzii, An. gambiae* and *An. arabiensis* (65.11, 24.21 and 7.39%, respectively). Similar to the results of the laboratory experiment, only spectra collected from *An. coluzzii* and *An. gambiae* were included in the data analysis. Spectra from these freshly scanned mosquitoes revealed a prediction accuracy of 88% (*An. gambiae*, 88%; *An. coluzzii*, 88%). After 2 weeks of preservation in silica gel, NIRS models trained on mosquitoes preserved in the same medium had a prediction accuracy of 84% (*An. gambiae*, 84%; *An. coluzzii*, 83%), whereas for those stored in 80% ethanol, NIRS was able to differentiate mosquito species with 79% accuracy (*An. gambiae*, 88%; *An. coluzzii*, 71%).

### Accuracy of NIRS to determine *Plasmodium* infection status of preserved *Anopheles* species

The proportions of *P. falciparum*-infected and -uninfected laboratory-reared *An. coluzzii* were 38.08% (139/365) and 61.92% (226/365), respectively. In comparison, the proportions of *P. falciparum*-infected and -uninfected field-caught *An. coluzzii* mosquitoes were 9.35% (43/460) and 90.65% (417/460). NIRS classified infected and uninfected laboratory-reared *Anopheles coluzzii* with an accuracy of 64% and 61% when in the fresh state and after preservation in silica gel, respectively. NIRS spectra from wild-caught mosquitoes also preserved in silica gel or ethanol were analyzed, and the prediction accuracy was low depending on the preservative (Table 3). Similar results were seen for both within-sample accuracy (predicting laboratory-infected mosquitoes using models calibrated on laboratory-infected mosquitoes) and out-of-sample accuracy (predicting wild-caught mosquitoes using models calibrated on laboratory-infected mosquitoes). NIRS had a poor predictive ability for differentiating infected and uninfected wild-caught mosquitoes irrespective of the preservation method (Table 3).

**Table 3:** Accuracy of near-infrared spectrometry to predict the infectious status of laboratory-reared, experimentally infected and wild *Anopheles coluzzii* based on two preservation procedures

Model trained on							Model predicting						
Mosquito	Killed	Preserved	Number	Within-sample accuracy (%)			Mosquito	Killed	Preserved	Number	Out-of-sample accuracy (%)		
				Accuracy	TPR	TNR					Accuracy	TPR	TNR
Laboratory-reared	Chloroform	Fresh	365	64	68	63	Laboratory	Chloroform	Silica gel	365	49	43	53
							Field	Chloroform	Fresh	460	50	49	50
		Silica gel	365	61	55	67	Laboratory	Chloroform	Fresh	365	52	52	52
							Field	Chloroform	Silica gel	327	51	60	43
Field-caught	Chloroform	Fresh all	460	56	61	51	Field	Chloroform	Silica gel	223	47	45	48
									Ethanol	237	52	51	52
		Silica gel	223	63	57	69	Field	Chloroform	Ethanol	237	53	45	62
		Ethanol	237	54	31	77	Field	Chloroform	Silica gel	223	54	38	70

Results are shown for overall accuracy, the true negative rate (TNR) and the true positive rate (TPR)

## Discussion

The potential for deployment of the spectroscopy technique in the field for monitoring mosquitoes and assessing malaria transmission is being considered. However, the question raised is “what would be the best method to collect mosquito samples given the limitations of some laboratory practices?” In the present study, we tested the Kaltox® insecticide, which is used in Burkina Faso for pyrethrum spray catches of mosquitoes for different research programs, for use in NIRS analysis. The results showed that pyrethrum spray catches could be used for NIRS species identification without interfering with the prediction accuracy. Indeed, In our study, NIRS accurately distinguished laboratory-reared *An. coluzzii* and *An. gambiae* s.s. independently of the killing option (pyrethrum spray with 90% accuracy vs chloroform with 92% accuracy), raising the prospect that this technology can be deployed using more practical mosquito collection methods for monitoring vector-borne diseases. This is the first study to address the question of using pyrethrum spray catches with encouraging results, although the results should be verified for other metrics of interest (such as determining mosquito age). In addition, mosquitoes killed with pyrethrum spray could be preserved in silica gel or 80% ethanol for 2 weeks for future analysis by the NIRS with good accuracy. Previous studies have already demonstrated that mosquitoes killed by chloroform can be stored under different conditions before NIRS scanning [12, 13, 17]. In our study, the accuracy of the NIRS technique (> 80%) for the identification of wild *Anopheles gambiae* s.l. species when silica gel was used as a preservative corroborates the results of these previous reports. In our study we used relatively small numbers of mosquitoes to calibrate the model, so we expect to achieve a better accuracy in terms of NIRS prediction by increasing the sample size, as shown in a previous work [22]. Interestingly, mosquito samples preserved in ethanol can be rehydrated using phosphate-buffered saline and dissected for other entomological study purposes after NIRS analysis.

*Anopheles* infection status for *Plasmodium* is one of the most important parameters when monitoring malaria transmission. A low accuracy of NIRS to predict the *Plasmodium* infection status of mosquitoes in the fresh state was observed in the present study as well as in previous studies [8, 9]. The accuracy of NIRS to predict *Plasmodium* infection status was still lower after preservation of the specimens in both 80% ethanol and silica gel. Because the difference in NIRS prediction of infection status did not differ between the fresh state and after mosquito preservation, we assume that this lower accuracy is not due to the preservation method. That NIRS does not work well in predicting the presence or absence of *Plasmodium* in wild mosquitoes irrespective of whether the models were trained on laboratory or field mosquitoes could be due to multiples factors, such as larval breeding site diversity, blood meal and sugar sources and physiological and nutritional status of the mosquito [9]. Further studies are needed to better explain the influence of preservation methods on NIRS accuracy to predict mosquito species or their *Plasmodium* infection status since only two methods (silica gel and 80% ethanol) were used in this 2-week-long study. Another limitation is the age of wild *Anopheles* in determining *Plasmodium* infection status. There is currently no gold-standard method for determining mosquito age in wild mosquitoes. In natural mosquito populations, older insects are more likely to be infected. While this variable could be accounted for in our laboratory experiment as the age of the mosquito was kept constant (3 days old), it could not be controlled in the wild mosquito experiment. It is currently unclear how well NIRS can determine mosquito age in wild mosquitoes. Nevertheless, future work should take into account the confounding factor of mosquito age when determining the sensitivity and specificity of diagnostic methods.

## Conclusion

Near-infrared spectroscopy has the potential to be a useful entomological surveillance tool for determining mosquito age, species and pathogen infection status, providing valuable data for control interventions aimed at malaria monitoring and evaluation. Numerous studies have demonstrated the promising potential of NIRS, but one of the main challenges remains identifying the best protocol for collecting the mosquito vectors. Our results show that mosquitoes can be killed by chloroform or pyrethrum spray and then analyzed by NIRS with high accuracy. NIRS classified both *An. gambiae* and *An. coluzzii* species killed using pyrethrum spray, implying that this insecticide and its derived molecules could be used as a practical alternative substance to kill mosquitoes for NIRS analysis. In addition, we found that while silica gel is the best preservative, in low-income countries where silica gel is expensive and/or not available, 80% ethanol can be used as an alternative to silica gel to preserve mosquito samples for future NIRS analysis. The possibility to preserve vector samples for future NIRS analysis offers the opportunities to centralize the technical aspects of NIRS in an appropriate laboratory, thereby saving costs. Despite these optimistic results on the method of killing and preserving mosquitoes for NIRS analysis, further studies are needed to screen more preservation methods that do not influence the accuracy of this technique.

## Supplementary Information

**Additional file 1.** Dataset S1.

**Additional file 2. **Dataset S2.

**Additional file 3: Figure S1.** Average spectra of laboratory-reared mosquitoes for each killing method: chloroform (blue) and Kaltox (red). Average spectra differed slightly between the two killing options.

**Additional file 4: Figure S2.** Average spectra of laboratory-reared mosquitoes for each killing and preservation method: fresh mosquitoes (blue) and preserved mosquitoes (red). Globally, a distinctive difference was observed between the average spectra from mosquitoes freshly analyzed compared to those obtained after *Anopheles* preservation in silica gel or in ethanol. However, mosquito NIRS spectra were influenced more by the silica gel than by ethanol.

## Data Availability

Additional file 1: Dataset S1; Additional file 2: Dataset S2.
