# Supplementary material for: Adapting field-mosquito collection techniques in a perspective of near-infrared spectroscopy implementation
Source: Parasit Vectors. 2022 Sep 26;15:338. doi: 10.1186/s13071-022-05458-6 (PMC9513905; doi:10.1186/s13071-022-05458-6)

Additional file 3: Figure S1: Average spectra of laboratory-reared mosquitoes for each killing method: chloroform (blue) and kaltox (red). The average spectra was slightly different between the two killing options.


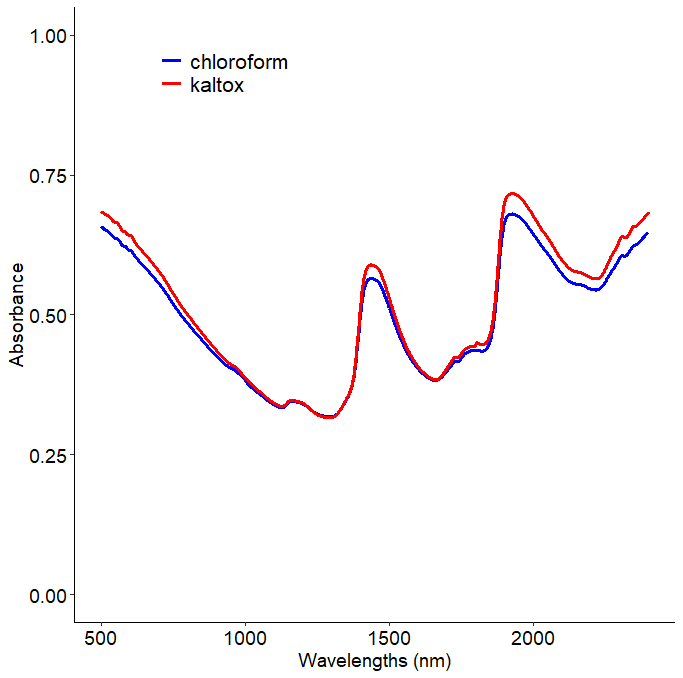

Supplement: Supplementary file 3 — Additional file 3: Figure S1. Average spectra of laboratory-reared mosquitoes for each killing method: chloroform (blue) and Kaltox (red). Average spectra differed slightly between the two killing options. [file 13071_2022_5458_MOESM3_ESM.docx]
