# Supplementary material for: Adapting field-mosquito collection techniques in a perspective of near-infrared spectroscopy implementation
Source: Parasit Vectors. 2022 Sep 26;15:338. doi: 10.1186/s13071-022-05458-6 (PMC9513905; doi:10.1186/s13071-022-05458-6)

Additional file 4: Figure S2: Average spectra of fresh laboratory-reared mosquitoes for each killing and preservation methods: fresh mosquitoes (blue) and preserved mosquitoes (red). Globally, a distinctive difference was observed between the average spectra from mosquitoes freshly analyzed compared to whom obtained after *Anopheles* preservation in silica gel or in ethanol. However, mosquito NIRS spectra were more influenced by the silica gel than ethanol.


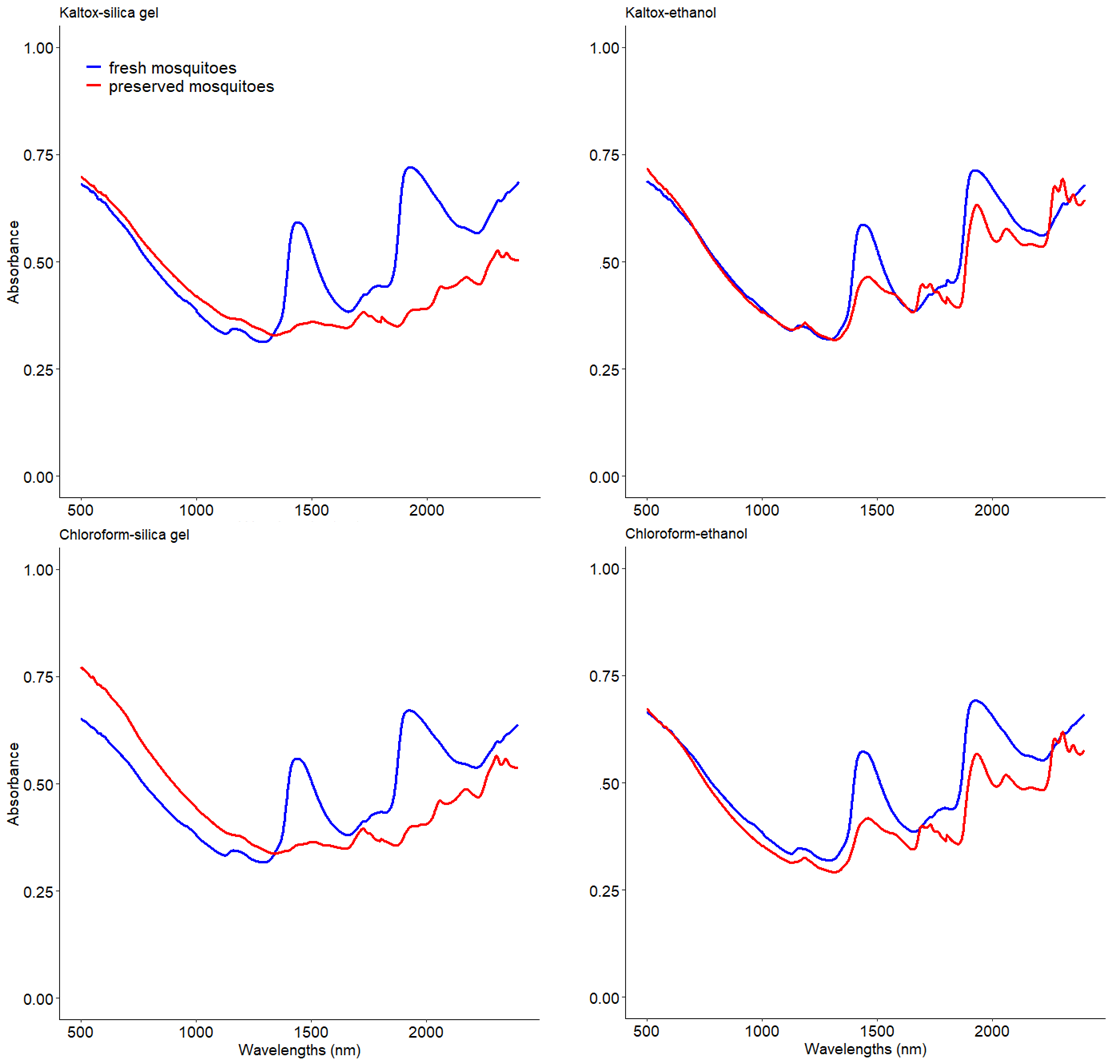

Supplement: Supplementary file 4 — Additional file 4: Figure S2. Average spectra of laboratory-reared mosquitoes for each killing and preservation method: fresh mosquitoes (blue) and preserved mosquitoes (red). Globally, a distinctive difference was observed between the average spectra from mosquitoes freshly analyzed compared to those obtained after Anopheles preservation in silica gel or in ethanol. However, mosquito NIRS spectra were influenced more by the silica gel than by ethanol. [file 13071_2022_5458_MOESM4_ESM.docx]
